# A novel molecular signature for predicting prognosis and immunotherapy response in osteosarcoma based on tumor-infiltrating cell marker genes

**DOI:** 10.3389/fimmu.2023.1150588

**Published:** 2023-04-06

**Authors:** Haijun Tang, Shangyu Liu, Xiaoting Luo, Yu Sun, Xiangde Li, Kai Luo, Shijie Liao, Feicui Li, Jiming Liang, Xinli Zhan, Qingjun Wei, Yun Liu, Maolin He

**Affiliations:** ^1^ Department of Spine and Osteopathic Surgery, The First Affiliated Hospital of Guangxi Medical University, Nanning, Guangxi, China; ^2^ Department of Pharmacy, The First Affiliated Hospital of Guangxi Medical University, Nanning, Guangxi, China; ^3^ Department of Radiotherapy, The Second Affiliated Hospital of Guangxi Medical University, Nanning, Guangxi, China; ^4^ Department of Orthopedics, The First Affiliated Hospital of Guangxi Medical University, Nanning, Guangxi, China

**Keywords:** osteosarcoma, tumor infiltrating lymphocytes, single-cell RNA sequencing, risk score, immunology

## Abstract

**Background:**

Tumor infiltrating lymphocytes (TILs), the main component in the tumor microenvironment, play a critical role in the antitumor immune response. Few studies have developed a prognostic model based on TILs in osteosarcoma.

**Methods:**

ScRNA-seq data was obtained from our previous research and bulk RNA transcriptome data was from TARGET database. WGCNA was used to obtain the immune-related gene modules. Subsequently, we applied LASSO regression analysis and SVM algorithm to construct a prognostic model based on TILs marker genes. What’s more, the prognostic model was verified by external datasets and experiment *in vitro*.

**Results:**

Eleven cell clusters and 2044 TILs marker genes were identified. WGCNA results showed that 545 TILs marker genes were the most strongly related with immune. Subsequently, a risk model including 5 genes was developed. We found that the survival rate was higher in the low-risk group and the risk model could be used as an independent prognostic factor. Meanwhile, high-risk patients had a lower abundance of immune cell infiltration and many immune checkpoint genes were highly expressed in the low-risk group. The prognostic model was also demonstrated to be a good predictive capacity in external datasets. The result of RT-qPCR indicated that these 5 genes have differential expression which accorded with the predicting outcomes.

**Conclusions:**

This study developed a new molecular signature based on TILs marker genes, which is very effective in predicting OS prognosis and immunotherapy response.

## 1 Introduction

Osteosarcoma (OS), the most common primary bone tumor in children and adolescents (1, 2), is characterized by high aggressiveness and poor prognosis (3). It is a relatively rare cancer with global incidence rate of approximately 4.8 per million per year (4, 5). Although treatments including surgery, chemotherapy and immunotherapy have made some progress in recent years, the overall survival rate is still unsatisfactory and fluctuates between 60% and 70% (6, 7). Therefore, it is particularly important to identify reliable prognostic markers and construct new molecular models to accurately predict the survival trend of patients with osteosarcoma.

The tumor microenvironment is a harbor for tumor cells and influences tumor development (8). Tumor infiltrating lymphocytes (TILs), one of the most common cell composition in tumor microenvironment, is composed of innate immune cells, adaptive immune cells, immunoreactive cells (e.g. cytotoxic T lymphocytes) and immunosuppressive cells (e.g. regulatory T cells) (9–11). Secrete cytokines derived from TILs can induce migration and aggregation of CXCR5-expressing B and T cells to suppress tumor progress. Chemotactic B cells and T cells form well-organized structures can prevent tumor metastasis to other sites (12, 13). In the context of antitumor immunotherapy, TILs are gradually gaining attention. In laryngeal squamous cell carcinoma, Sara et al. concluded that the number of TILs was positively correlated with PD-L1 expression and a good prognosis (14). In colorectal cancer, Yu-jie Liang et al. concluded that patients with high levels of Foxp3+ T cells had a better prognosis (15, 16). Thus, TILs could be used as biomarkers with good prognostic predictive value in a variety of tumors (12, 17). Although some prognosis gene models of OS have been established (18), few studies have reported and developed a prognostic risk model based on TILs in OS.

We firstly found TILs marker genes based on single cell RNA sequencing (scRNA-seq) data. Subsequently, in order to screen the prognostic genes and established risk model, LASSO regression analysis and machine learning SVM algorithm were utilized. Ultimately, we developed a prognostic risk model successfully, which may offer a novel reference for predicting the prognosis of OS patients.

## 2 Materials and methods

### 2.1 Preliminary experiment and raw data acquisition

ScRNA-seq sequencing data was obtained from our previous research, in which we collected tumor tissue from 6 OS patients from our hospital and conducted single-cell RNA sequencing (19). The basic information of the patients was showed in **Table S1**. Tumor tissues collecting from surgery were cut into pieces of approximately 1 mm^3^ in size and converted into cell suspensions for use. The sequencing work was performed in a double-end sequencing mode and i.e. 150 bases were measured at both Read 1 and Read 2 ends. We followed the 10X Genomics’ official process to perform upstream analysis using Cell Ranger software (version 4.0.0). We aligned the single-cell data with the human genome sequencing reference library GRCh38. The barcode.tsv file, gene.tsv file and matrix.mtx file were then derived by pairing read lengths, generating feature barcode matrices, performing clustering and other secondary analyses (19, 20). Furthermore, the data used for prognostic signature model development were from TARGET databank (https://ocg.cancer.gov/programs/target). Two external verification databases named GSE21257 and GSE16091 were downloaded from Gene Expression Omnibus (GEO) database (https://www.ncbi.nlm.nih.gov/geo).

### 2.2 Identification of TILs marker genes

In this study, single-cell RNA sequencing data was analyzed by “Seurat” and “SingleR” R packages (21). In order to seek osteosarcoma cells, we rigorously filtered the raw matrix data of each cell based on three filtering criteria: nFeature_RNA > 300, nFeature_RNA < 4500 and percent. mt < 15. First, we set the mode to “LogNormalize” when we normalized the data through “NormalizeData” function of the “Seurat” R package. The normalized data was then transformed into a Seurat object, which was processed by “FindVariableFeatures” function to identify the genes of ideal cells. Next, “RunPCA” function of the “Seurat” R package analyzes the top 2000 highly variable genes of the target cells. The result of PCA were presented as PCA scatter plots. The top 30 principal components (PCs) were identified by JackStraw analysis. The cell clustering analysis was performed by using “FindNeighbors” and “FindClusters” functions of the “Seurat” R package. The clustering results were visualized as t-distributed random neighborhood embeddings (t-SNE) by “RunTSNE” function. We used “FindAllMarkers” function of the “Seurat” R package to identify differentially expressed genes (DEGs) for each cluster according to the criteria of adjusted p < 0.05 and |log2(FC)| >0.25. The “SingleR” R package was leveraged to pair the DEGs with marker genes from various cell types in the human primary cell atlas, thus enabling the annotation of cell clusters. Besides, we implement metabolomic analysis of TILs with “scMetabolism” R package (22).

### 2.3 Immune-related co-expression analysis

We first extracted the expression profile data of TILs marker genes from TARGET cohorts which includes 85 RNA expression matrix. On the basis of expression profile data of TILs marker genes, we calculated tumor-associated stromal scores, immune scores, ESTIMATE scores and tumor purity using “estimate” R package (23). We then used “WGCNA” R package to find the modules that were most significantly positively correlated with stromal scores, immune scores and ESTIMATE scores but most significantly negatively correlated with tumor purity. These genes included in the selected modules were defined as immune-associated TILs marker genes and used in the construction of a prognostic signature model.

### 2.4 Construction of a prognostic signature based on TILs marker genes

We first integrated the expression profile data of immune associated TILs marker genes with the clinical phenotype data of 85 TARGET cohorts, and then used univariate Cox regression analysis to screen for genes with prognostic value. In the LASSO regression analysis implemented by “glmnet” R package, we used “cv.glmnet” function to perform a 10-fold cross-validation of prognosis genes and then obtained genes with non-zero β coefficients. Simultaneously, according to the survival status, patients in the TARGET cohorts were assigned into survival or death group. In the machine learning SVM regression analysis, “svmRadial” function of the “e1071” R package was used to cross-verify the prognostic genes and aggregate the genes with the lowest error. The genes obtained from both analysis methods were intersected and common genes were extracted. The multivariate Cox regression analysis was the final step in creating the TILscore and assessing risk. The risk score of TILScore was calculated based on the equation “risk score = Σexpgenei*βi”. The “expgene” is the expression value of the model gene and “β” is the risk coefficient of the model gene. Median risk score splitted OS patients of the TARGET cohorts into two risk groups.

### 2.5 Validation of prognostic signature based on immune-related TILs marker genes

To evaluate the prognostic performance of TIL score, we used “ survivalROC” R package to obtain time-dependent ROC curves (24), and area under curve (AUC) values reflect the predictive ability of the model for patients’ overall survival at 1, 3, and 5 years. We invoked “survminer” R package in the Kaplan-Meier survival analysis to investigate survival differences between the high-risk and low-risk groups. In addition, we examined the possibility of TILScore as an independent prognostic factor by independent prognostic analysis. We combined TILScore with other clinical phenotypes to form a clinical nomogram. The clinical nomogram initially predicted the prognosis of different patients at 1,3,5 years.

In order to further verify the reliability of the model, we collected the expression matrix and clinical information from two GEO databases (GSE21257 and GSE16091). After removing the batch effect, the two databases were merged by Combat function of “sva” package. Subsequently, the merged database will be used as external verification database.

### 2.6 Tumor immune landscape assessment and immunotherapy response prediction

First, we extracted the expression profiles of 2044 TILs genes with cell type identification by estimating relative subsets of RNA transcripts (CIBERSORT) algorithm and gained the infiltration abundance of 22 immune cell types. Subsequently, we analyzed the proportion of immune cell infiltrates in the two risk groups. In addition, we performed differential analysis of the expression matrix data of the immune checkpoint genes and observed the expression levels between the two risk groups.

### 2.7 Function and pathway enrichment analysis

In this study, we performed Genes Ontology (GO) and Kyoto Encyclopedia of Genes and Genomes (KEGG) analyses with “clusterProfiler” R package (25). We used “clusterProfiler” R package to visualize the functions and pathways of TILs marker genes. The GO annotations are based on the genome-wide annotation package released by the Bioconductor project (org.Hs.eg.db). The KEGG annotations were queried through the web API in the “clusterProfiler” R package for the latest online KEGG database. P value <0.05 was considered as a significant enrichment criteria.

### 2.8 Cell cultures

In this study, human osteoblasts cell (OB) were used as a control group, while human OS cells including 143B and HOS cells as experimental cell. All cells were purchased from Procell Life Science&Technology Co.,Ltd. (Wuhan, China). 143B cells and HOS cells were cultured in 1640 medium (Gibco, USA) and MEM medium, respectively. The two medium was supplemented with 1% penicillin/streptomycin (Solarbio, Beijing, China) and 10% fetal bovine serum (FBS; Gibco). OB cells cultured in DMEM/F-12 medium. OS cells were cultured in a humidified 5% CO2 incubator at 37 °C while OB cells was in a humidified 5% CO2 incubator at 35 °C.

### 2.9 RT-qPCR assays

In accordance with manufacturer’s instructions, total RNA was extracted using RNA fast 200 Kit (Fastagen Biotech, Shanghai, China). RNA was reverse-transcribed into complementary DNA (cDNA) by using a cDNA synthesis kit (Takara, Japan). RT-qPCR was performed using SYBR Green (FastStart Universal SYBR Green Master ROX, Germany) on a StepOnePlus™ Real-Time PCR System (ABI7500). The PCR procedure was as follow: 95 °C for 10 minutes, followed by 40 cycles at 95 °C for 10 seconds and 60 °C for 1 minute. The gene expression level in cell lines was expressed as relative expression and calculated using the ^2-ΔΔ^Ct method. The primer sequences can be found in **Table S4**.

### 2.10 Statistical analysis

In this study, data was analyzed and generated using R software version 4.1.0 (http://www.R-project.org). P < 0.05 was the significance threshold and the “p.ucdilg” R function was used to adjust the P values for multiple analyses.

## 3 Results

### 3.1 Identification and annotation of TILs clusters

**Figure 1:**
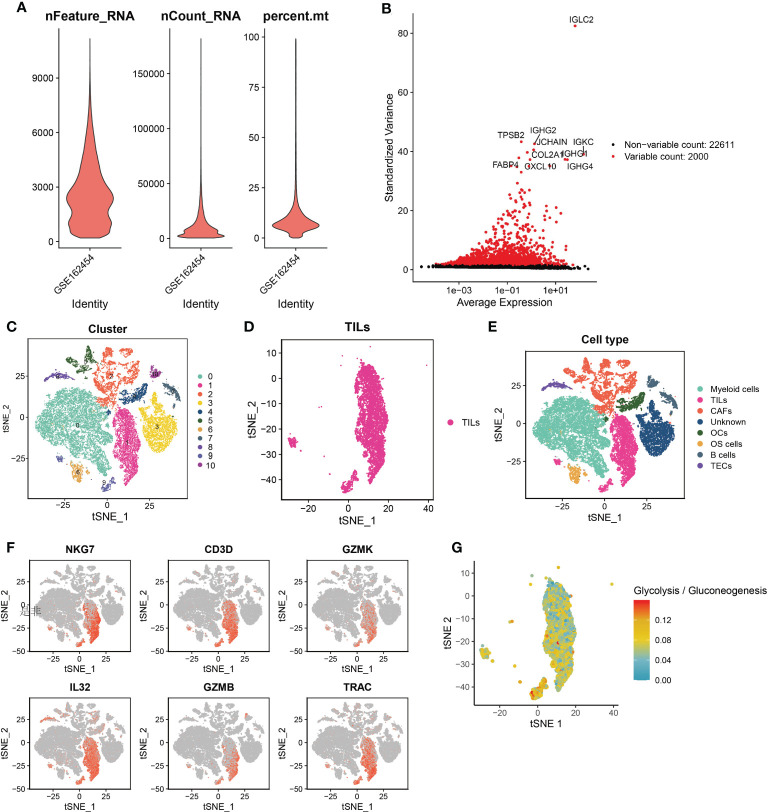
Quality control of cells and the single-cell RNA sequencing to identify TILs markers. **(A)** Violin plot of 3 cell quality control standards. **(B)** The top 2,000 high variance genes. **(C)** t-SNE plots sorted by cell clusters. **(D)** t-SNE plots colored by the same cell type. **(E)** Different cell clusters identified by marker genes. **(F)** TILs identified by 6 marker genes. **(G)** single cell metabolic features of TILs are related to glycolysis or gluconeogenesis.

First, quality control criterias (nFeature_RNA > 300, nFeature_RNA < 4500, %. mt < 15) were used to filter single-cell RNA sequencing data of 6 patients with osteosarcoma. We obtained 24,611 genes from high quality cells and selected the top 2,000 high variance genes (**Figures 1A, B**). Next, we incorporated these 2000 high-variance genes into PCA analysis to reduce the dimensionality of the sequencing data. The cells were then subjected to co-expression clustering analysis to obtain 11 cell clusters (**Figure 1C**). Subsequently, each cluster were annotated by automatically coordinating the “SingleR” R package and manually based on marker genes. We defined the cells of cluster 1 and cluster 9 as TILs with the marker genes NKG7, CD3D, GZMK and GZMB (**Figures 1D–F**). After integrating the data of cluster 1 and cluster 9, a total of 2078 marker genes were obtained. In addition, we found that the single cell metabolic features of TILs are related to glycolysis or gluconeogenesis (**Figure 1G**).

### 3.2 Functions and pathways of TILs marker genes

**Figure 2:**
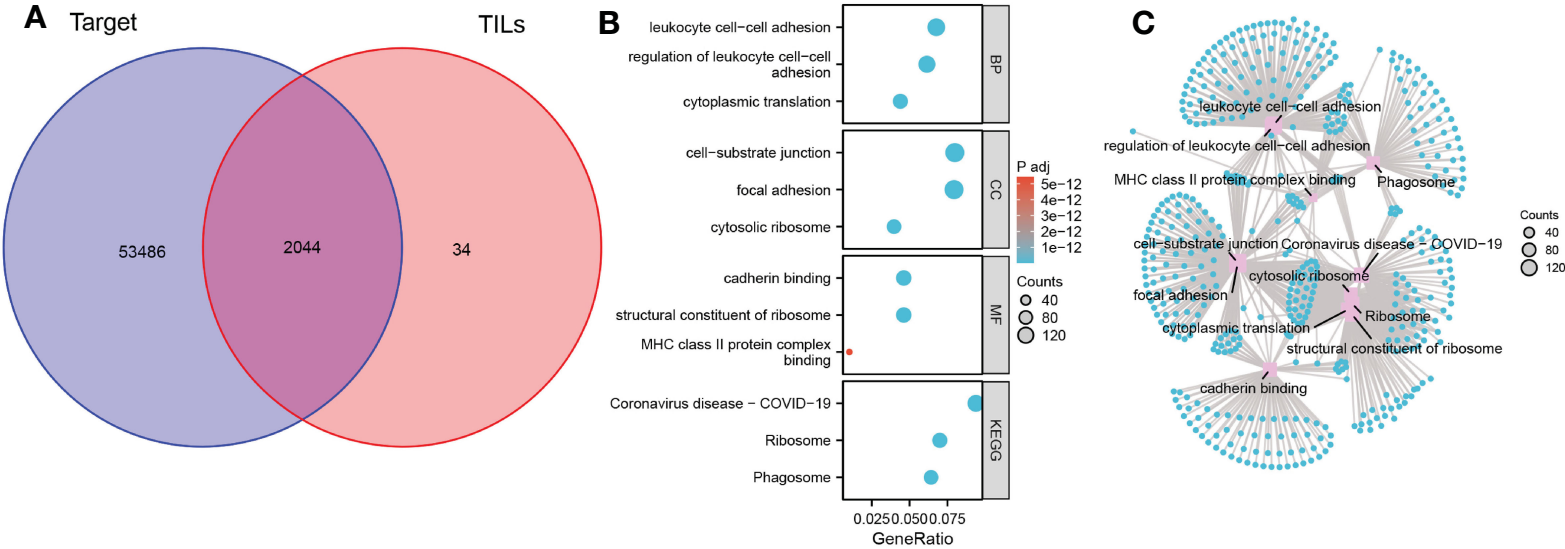
Biological functions and pathways of TILs marker genes. **(A)** Venn diagram of 2044 TILs marker genes from TARGET OS cohort and scRNA-seq data. **(B)** Bubble plot of the functions and pathways of TILs marker genes. **(C)** Network plot of the functions and pathways of TILs marker genes.

Enrichment analysis was used to understand the role of TILs in the anti-tumor immune response. First, we used the previous 2078 TILs marker genes in combination with genes expression matrix data from 85 TARGET cohorts to obtain expression profile data for 2044 marker genes (**Figure 2A**). The results of GO analysis showed that these marker genes are mainly involved in the biological processes of immune cell adhesion, cell migration and protein translation, including regulation of leukocyte cell-cell adhesion, focal adhesion, cell-substrate junction and cadherin binding. The KEGG analysis also confirmed the close association of these genes with immune cell adhesion and ribosomes (**Figures 2B, C**)

### 3.3 Establishment of a prognostic signature on the basis of five TILs marker genes

**Figure 3:**
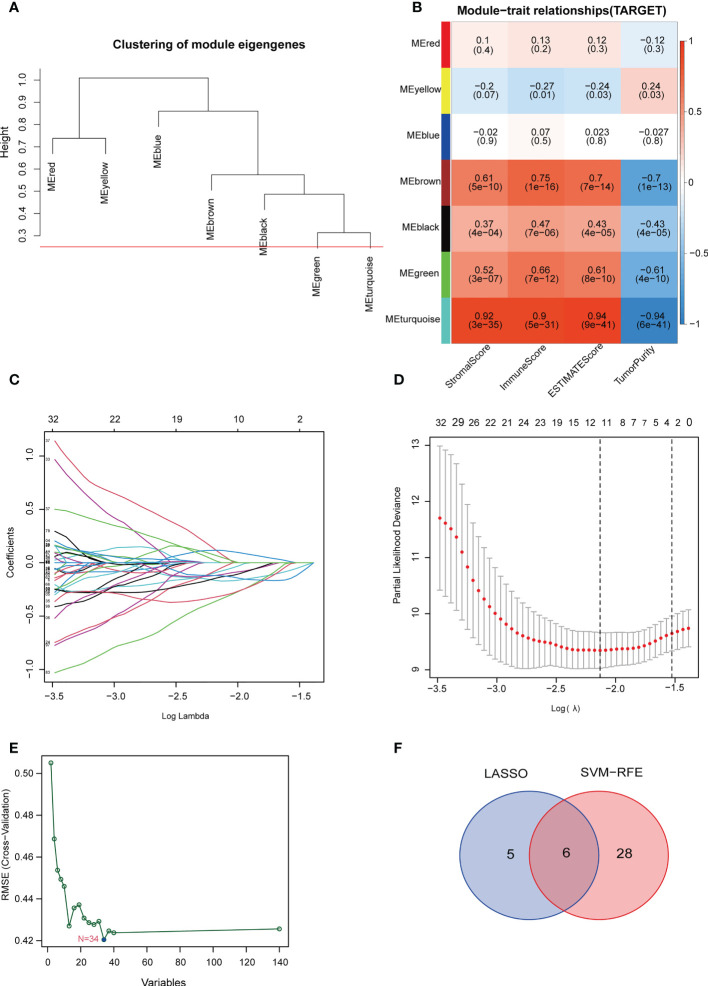
Construction of the TILscore. **(A)** Module thresholds for WGCNA analysis. **(B)** Coefficients of different modules showing the correlation of TILs marker genes with 4 microenvironment scores. **(C, D)** Coefficient and parameter plots of LASSO analysis showing the 11 filtered candidate genes and pathways of TILs marker genes. **(E)** Machine learning SVM analysis curve showing 34 filtered candidate genes. **(F)** Venn diagram of common candidate genes from LASSO analysis and machine learning SVM analysis.

The optimal soft threshold was set to 7 and the results of WGCNA analysis showed that a total of 7 gene modules were identified. The 545 genes in the blue-green module were most significantly positively correlated with stromal scores, immune scores, and ESTIMATE scores, but most significantly negatively correlated with tumor purity (**Figures 3A, B**). Thus, the genes in the blue-green module are marker genes associated with immunity. Next, we performed a univariate Cox regression analysis on 545 marker genes and found that 140 genes were significantly associated with overall survival (**Figure S1**). Subsequently, we screened 11 genes and 34 genes from the 140 prognostic genes using LASSO Cox regression analysis and machine learning SVM regression analysis, respectively (**Figures 3C-E** and **Table S2**). The genes obtained from the two analysis methods were taken to intersect to obtain 6 common genes (**Figure 3F** and **Table S2**). After multivariate Cox regression analysis, 5 genes were remained and used to construct a prognostic signature model. We also performed risk assessment based on the model. In addition, the risk score of the prognostic model = (0.705 × EPHX2 expression value) + (0.478 × FDPS expression value) + (-0.35 × GBP1 expression value) + (-0.726 × MMD expression value) + (-0.815 × ZYX expression value) (**Table S3**).

### 3.4 Survival analysis of TILScore

**Figure 4:**
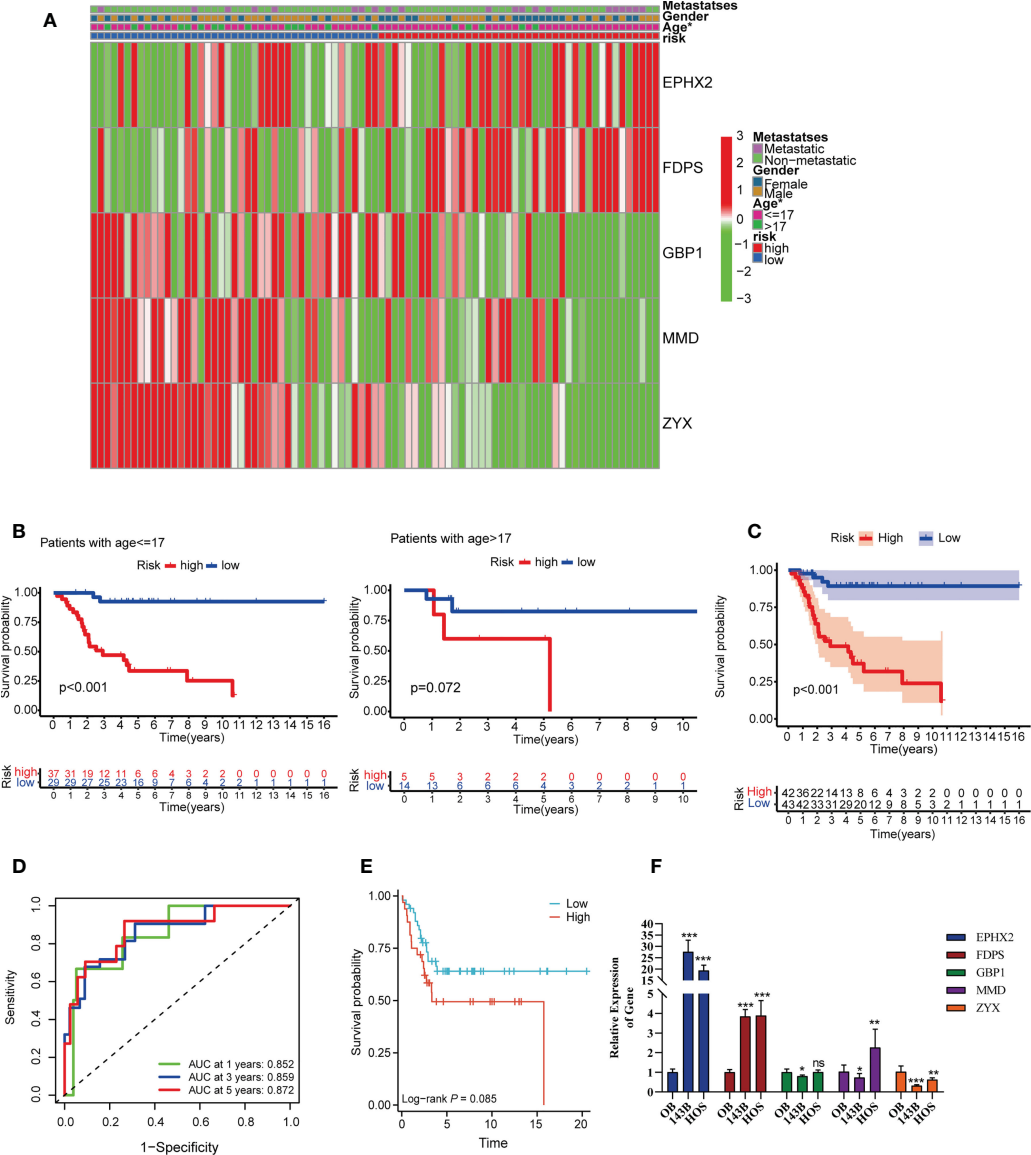
Survival analysis and predictive performance evaluation of TILscore. **(A)** Heat map of the relationship between different clinical phenotypes and two risk groups. **(B)** 17 years of age as the threshold for survival differences between the two risk groups. **(C)** Kaplan-Meier curves for survival analysis compared overall survival of OS patients in the high-risk and low-risk groups. **(D)** TILscore ROC curves predict the risk of death at 1, 3, and 5 years. **(E)** Kaplan-Meier curves for survival analysis based on external verification database. **(F)** The result of RT-qPCR. *p < 0.05; **p < 0.01; ***p < 0.001; ns is for no statistical significance.

Their median value of the risk score was 0.874 and categorized the patients in TARGET cohorts into a low risk group (n = 43) and a high risk group (n = 42). **Figure 4A** showed the relationship among risk scores, clinical phenotype, and modeled genes. Risk model are all had prognostic value in different sub-groups except the group of age (**Figure 4B** and **Figure S2**). Kaplan-Meier analysis also showed a more advantageous overall survival rate in the low-risk group (**Figure 4C**). The AUC values of the ROC analysis were 0.852, 0.859, 0.872 at 1, 3, 5 years, which indicated the high predictive accuracy of the prognostic signature model (**Figure 4D**). **Figures S3A, B** showed the survival status of patients with different risk scores. Independent prognostic analysis using “age, sex, tumor metastasis, TILScore” revealed that TILScore (HR: 1.244,95% CI: 1.149-1.347, P<0.001) and tumor metastasis (HR: 4.691,95% CI: 2.160-10.187, P<0.001) were independent prognostic factors (**Figures S4A, B**). Finally, nomograms was constructed to predict the survival state at the 1st, 3rd, and 5th years (**Figures S4C, D**). To verify the reliability of the model, we combined two GEO datasets and ultimately obtained 82 examples. According to the quartile method, patients were divided into two groups by the risk score calculated by our formula. The result showed that patients in high risk group had low overall survival rate (p = 0.085) (**Figure 4E**). What’s more, the result of RT-qPCR released that, compared to OB cells, EPHX2 and FDPS were significantly overexpressed in 143B and HOS cells, while the expression of ZYX and GBP1 was significantly lower in 143B and HOS cells. The expression trend of MMD was not definite (**Figure 4F**). In conclusion, we successfully established a 5-gene TILScore based on TILs marker genes.

### 3.5 Immune landscapes associated with TILscore

**Figure 5:**
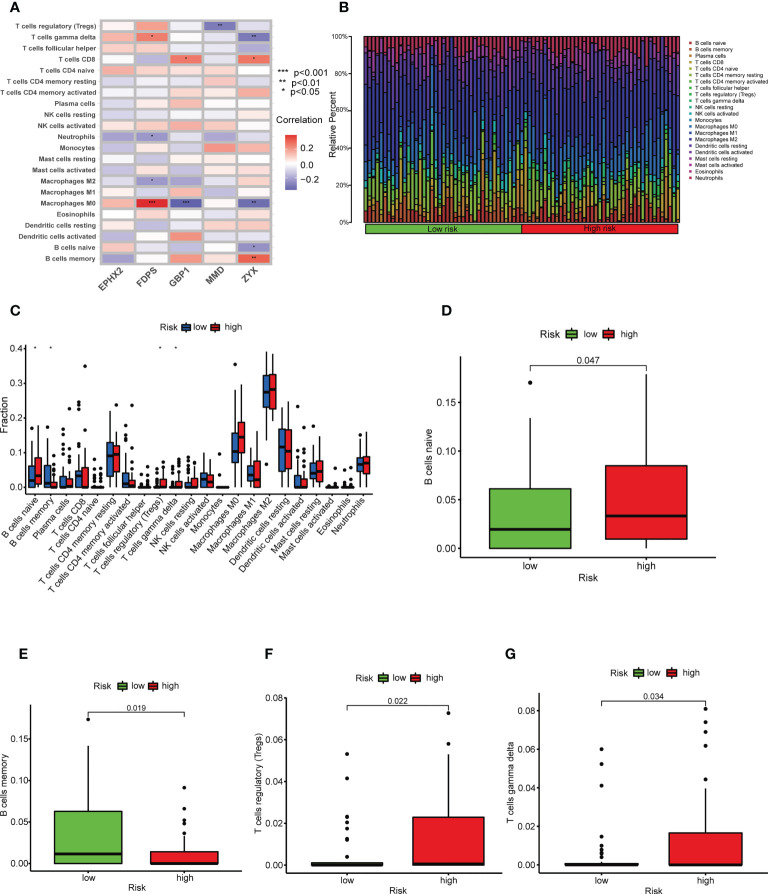
TILscore-associated immune landscape. **(A)** Heat map of the correlation between the 5 genes of TILscore and the abundance of 22 immune cell infiltrates. **(B)** Heat map showing the infiltration abundance of 22 immune cell species in different risk groups. **(C)** Box plots showing differences in immune cell infiltration by risk group. Differences among naive B cells **(D)**, memory B cells **(E)**, Treg cells **(F)** and gamma delta T cells **(G)** in the high-risk and low-risk groups. *p < 0.05; **p < 0.01; ***p < 0.001.

Because TILs are critical in the antitumor immune response, we explored the relationship between TILScore and immune cell infiltration. Differential analysis of four tumor-associated microenvironment score revealed that high-risk patients had lower immune scores, stromal scores and ESTIMATE scores (**Figures S5A, B**). Thus, the high-risk score was negatively correlated with the level of immune cell infiltration. The CIBERSORT algorithm calculated associations between five modeled genes and 22 immune cells (**Figure 5A**). The differences in the infiltration abundance of memory B cells, naive B cells, Treg cells, and γδ T cells were statistically significant in two risk groups (**Figures 5B, C**). Details of the infiltration of the four immune cells mentioned above were shown in **Figures 5D-G**. Genes of the prognostic signature may further affect the development of osteosarcoma by influencing the antitumor response through B and T cells.

### 3.6 The TILScore predicts immunotherapy benefits in OS patients

**Figure 6:**
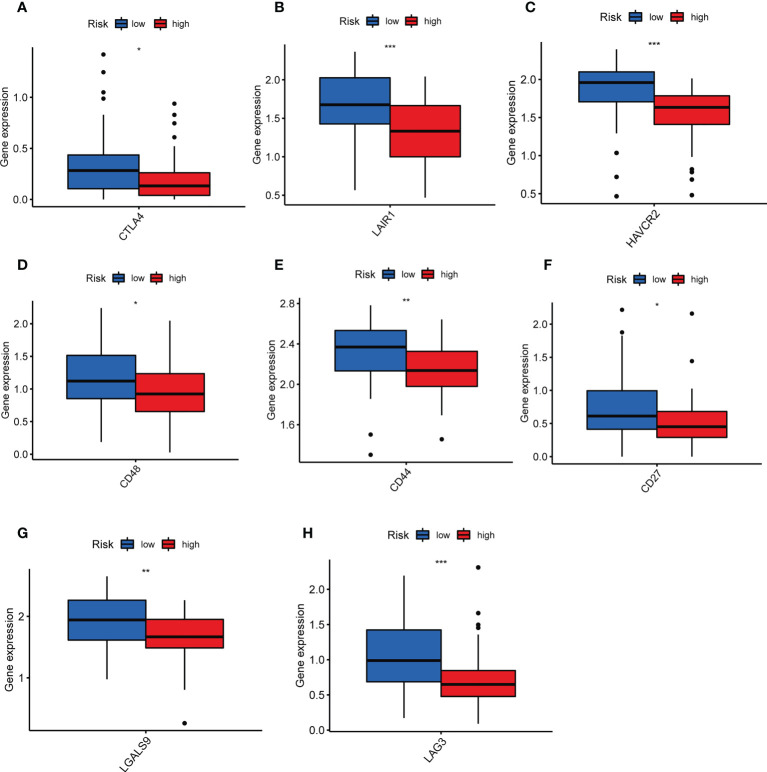
Differential analysis of immune checkpoints to assess the predictive power of TILscore immunotherapy. Differences in expression levels of immune checkpoints CTLA4 **(A)**, LAIR1 **(B)**, HAVCR2 **(C)**, CD48 **(D)**, CD44 **(E)**, CD27 **(F)**, LGALS9 **(G)**, and LAG3 **(H)** in the low- and high-risk groups. *p < 0.05; **p < 0.01; ***p < 0.001.

TILs are involved in antitumor immune responses, so we analyzed the relationship between TILScore and immune checkpoints. The results revealed that some critical immune checkpoints, such as CTLA4, LAIR1, HAVCR2, CD48, CD44, CD27, LGALS9 and LAG3, possess higher expression in low-risk group (**Figures 6A-H**).

## 4 Discussion

Previous studies have demonstrated that tumor-infiltrating lymphocytes are strongly associated with the prognosis of different tumor types (26–28), whereas few prognosis models have been conducted in osteosarcoma. In recent years, by using scRNA-seq analysis, Peng Song et al. developed a prognostic signature based on NK cell marker genes in patients with lung adenocarcinoma (29), suggesting that scRNA-seq analysis is an excellent tools to the construction of prognosis model. Therefore, we searched for marker genes of TILs by scRNA-seq analysis in present study. Our results showed that TILScore proved to be a powerful predictive model for OS patients’ prognosis. GO and KEGG analysis turned out that most of these TILs genes were enriched in biological processes such as immune cell adhesion, cell migration and protein synthesis pathways. Therefore, these genes may influence the proliferation and developmental processes of osteosarcoma through immune cell migration and cellular protein synthesis (30, 31).

Our risk model consisted of five TILs marker genes (EPHX2, FDPS, GBP1, MMD and ZYX). FDPS, an osteoclast farnesyl pyrophosphate synthase, is closely associated with osteosarcoma formation. Its main role is to promote osteoclast bone resorption activity and to inhibit osteoclast apoptosis (32). Upregulation of FDPS expression is also significantly associated with patients’ poor prognosis (33). MMD, one of the proteins associated with monocyte-to-macrophage differentiation, can enhance ERK1/2 and Akt phosphorylation in macrophages after LPS stimulation. It is involved in the antitumor immune response (34). Consistent with our findings, this gene is closely associated with phagosomes of tumor-infiltrating lymphocytes. Higher expression levels of MMD were found in the low risk group of this study and MMD was associated with good prognosis. ZYX is a LIM structural domain protein, which is involved in cytoskeletal organization and tumorigenesis (35, 36). ZYX is involved in apoptosis and osteoblast differentiation processes (37, 38). Therefore, ZYX may be involved in the development of osteosarcoma. High expression levels of ZYX were associated with good prognosis. EPHX2, the coding gene of soluble epoxide hydrolase (sEH) protein, is related to activity of epoxide hydrolase and phosphatase (39). EPHX2 was demonstrated to be strongly associated with cancer prognosis and macrophage phagocytosis (40). GBP1, encoding Guanylate Binding Protein 1, is related to tumor progression and chemotherapy drug resistance (41). What’s more, GBP1 was also regarded as microbe-specific gatekeeper of macrophage apoptosis and pyroptosis (42). Combining literature reports and our results of bioinformatics and RT-qPCR, we hold that EPHX2 and FDPS are the oncogenes, while GBP1, MMD and ZYX are the anti-oncogene in osteosarcoma. Risk model based on these five genes is very reliable to predict the prognosis of patients.

The differences in the immune landscape between risk groups allowed us to see the potential value of TILScore in predicting immunotherapy response. In the present study, naive B cells, Treg cells and γδ T cells were more distributed in the high-risk group, but memory B cells were more distributed in the low-risk group. Naïve B cells are undifferentiated B cells without antigen stimulation and infiltrate a high percentage in tumor tissue (43). Memory B cells are generated in the germinal center response during T cell-dependent immune response. Unlike naïve B cells, memory B cells are involved in a faster and stronger immune response (44). Therefore, the antitumor immune responses of memory B cells are faster, thus the prognosis of the low-risk group is better. Previous studies confirmed that Treg cells are involved in the tumor development process by suppressing antitumor immunity and its high expression level represents a poor prognosis (45). γδ T cells, a congenital cytotoxic T cells, were highly infiltrated in the low-risk group, which may be related to the fact that osteosarcoma is an immunocompromised tumor (46). Moreover, LAIR1, HAVCR2, CD27, CTLA4, CD48, CD44, LAG3 and LGALS9 were highly expressed in the low-risk group, indicating that the low-risk group was more likely to benefit from more types of immunotherapy. In summary, TILScore, a reliable biomarker for predicting response to immunotherapy, predicting that low-risk patients were more likely to benefit from immunotherapy.

There are some limitations of our study. First, a full functional experiment to elucidate the specific mechanisms of TILs marker genes in osteosarcoma is still very important. Second, the data involved in our study were derived from single-cell RNA sequencing data and the TARGET database, but the sample size remains insufficient. Therefore, the predictive power of this prognostic feature has some limitations.

## 5 Conclusion

Overall, this study developed a 5-gene prognostic signature based on TILs marker genes, which performed well in predicting prognosis and immunotherapy response in patients with osteosarcoma. TILScore can be considered to be an independent prognostic factor to predict patient prognosis and to guide patients to benefit from different immunotherapy methods.

## Data availability statement

The original contributions presented in the study are included in the article/**Supplementary Material**, further inquiries can be directed to the corresponding author/s.

## Ethics statement

The studies involving human participants were reviewed and approved by Ethics Committee and Institutional Reviewer Board of the First Affiliated Hospital of the Guangxi Medical University. Written informed consent to participate in this study was provided by the participants’ legal guardian/next of kin.

Written informed consent was obtained from the individual(s), and minor(s)’ legal guardian/next of kin, for the publication of any potentially identifiable images or data included in this article.

## Author contributions

All authors contributed to the article and approved the submitted version.

## Supplementary material

Supplementary Figure 1 Forest plot showing 140 prognostic genes screened by univariate Cox regression analysis.

Supplementary Figure 2 Survival differences between the two risk groups in subgroups of other clinical indicators. **(A, B)**: gender group; **(C, D)**: metastatic group.

Supplementary Figure 3 The distribution of risk score **(A)** and survival status **(B)**.

Supplementary Figure 4
**(A, B)** TILscore and tumor metastasis are independent prognostic factors. **(C, D)** Nomogram for predicting survival status of OS patients at the 1st, 3rd and 5th year.

Supplementary Figure 5 Visualization of differences in microenvironment scores across risk groups. **(A)** Heat map showing the difference between the 4 microenvironment scores in the high and low risk groups. **(B)** Box plots of differences in basal scores, immune scores, and ESTIMATE scores across risk groups.
