# Supplementary material for: A novel molecular signature for predicting prognosis and immunotherapy response in osteosarcoma based on tumor-infiltrating cell marker genes
Source: Front Immunol. 2023 Apr 6;14:1150588. doi: 10.3389/fimmu.2023.1150588 (PMC10117669; doi:10.3389/fimmu.2023.1150588)
Supplement: Supplementary file 1 [file DataSheet_1.docx]

Supplementary Material

# Supplementary Figures and Tables

## Supplementary Figures


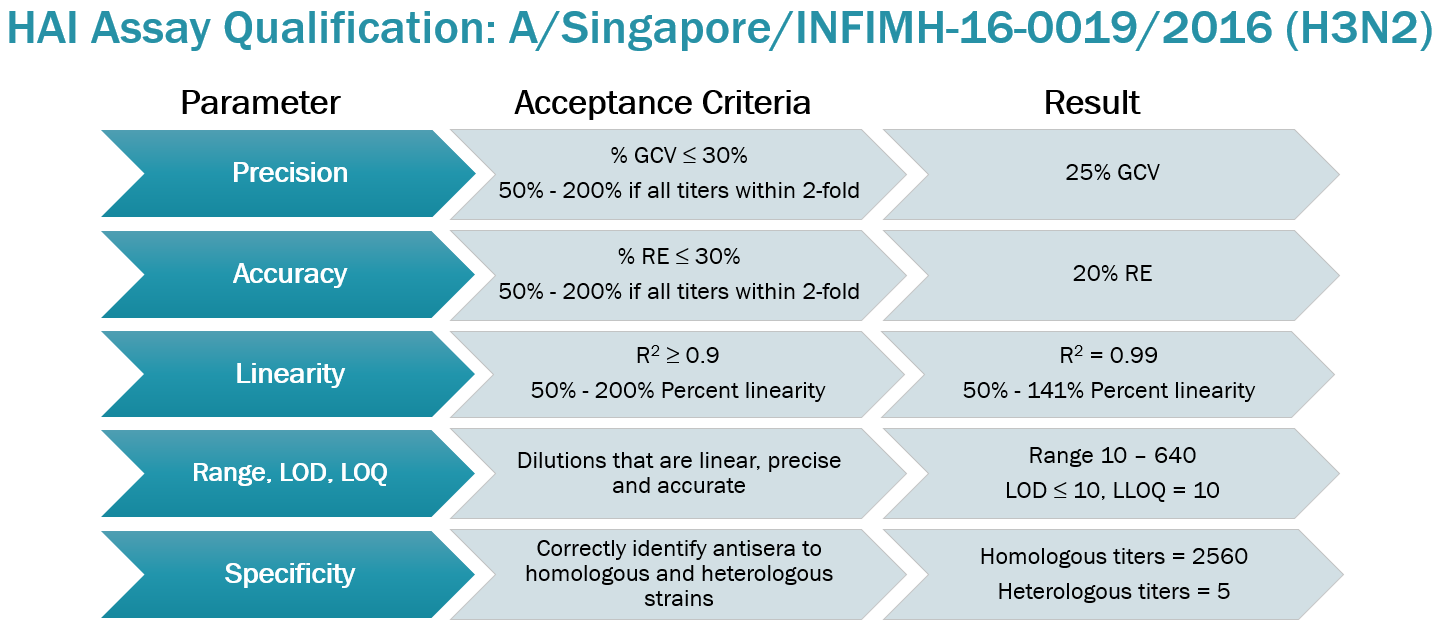


**Supplementary Figure 1: HAI assay extended qualification for H3N2 Influenza Strain A/Singapore/INFIMH-16-0019/2016.** Overview of qualification plan and parameters tested during extended qualification, pre-set acceptance criteria, and the results.


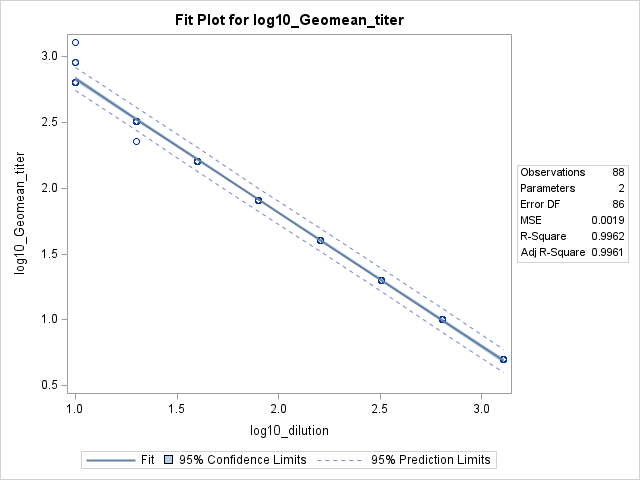


**Supplementary Figure 2: HAI assay extended qualification for H3N2 Influenza Strain A/Singapore/INFIMH-16-0019/2016, linearity testing results.** The plot shows SAS output from REG procedure, showing the linear regression analysis between log10 GMT [plotted on y-axis] and log10 dilution [plotted on x-axis]. The resulting R^2^ and R was 0.99.


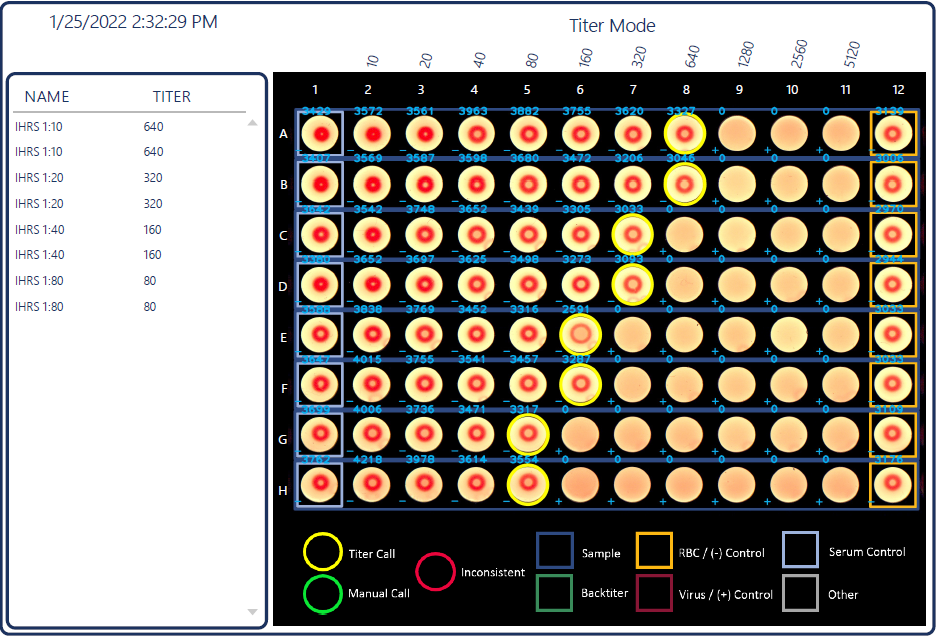


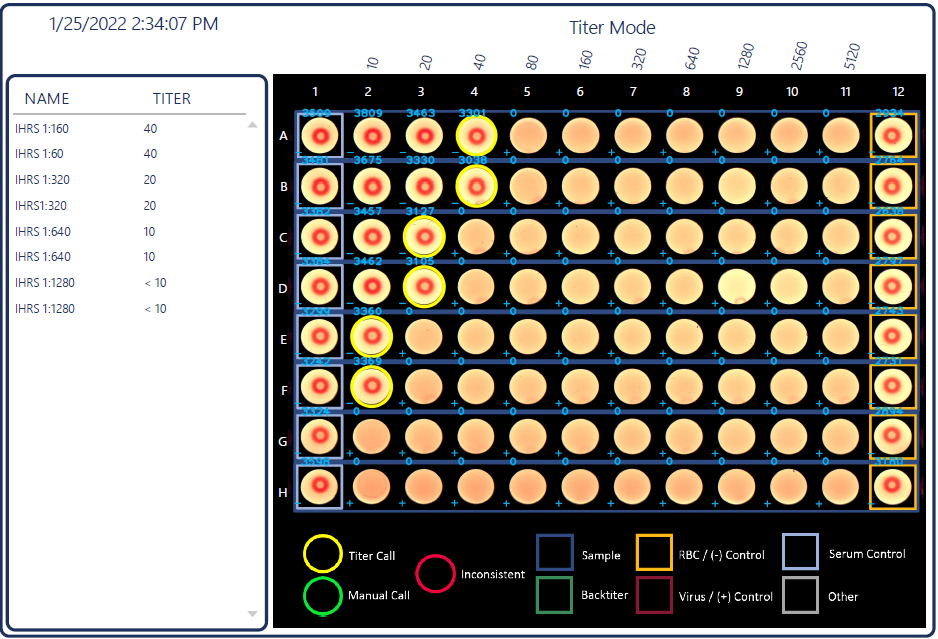


**Supplementary Figure 3: HAI assay extended qualification for H3N2 Influenza Strain A/Singapore/INFIMH-16-0019/2016, linearity testing plate visual.** A CypherOne instrument graphic demonstrating assay linearity, from a representative assay. The HAI titer decreased two-fold for each two-fold decrease in analyte concentration.


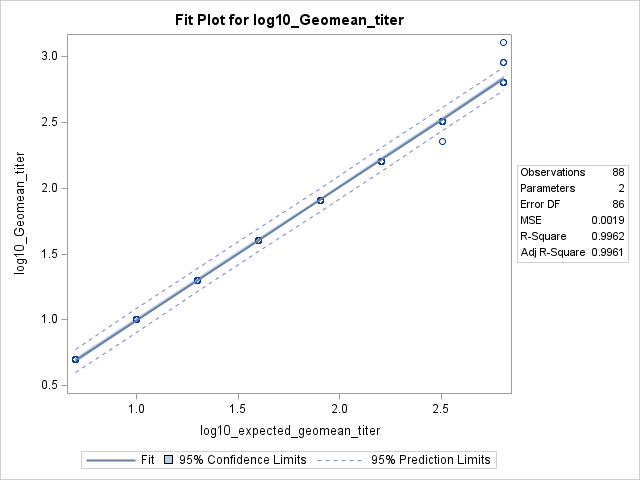


**Supplementary Figure 4: HAI assay extended qualification for H3N2 Influenza Strain A/Singapore/INFIMH-16-0019/2016, range testing results.** The plot shows SAS output from REG procedure, showing the linear regression analysis between the expected [plotted on x-axis] and observed log10 GMTs [plotted on y-axis]. The resulting R^2^ and R was 0.99.

## Supplementary Tables

**Supplementary Table 1: HAI assay extended qualification for H3N2 Influenza Strain A/Singapore/INFIMH-16-0019/2016, Systems Suitability Criteria testing.** GMTs, of samples tested for SSC, by sample description, assay plate number and assay identifier, and expected values for the respective samples. Results from two scientists, each performed two assays each, resulting in a total of four assays, and three plates within in each assay.

| Obs | Sample Description | Plate number | EQ_SSS Assay1 | EQ_SSS Assay2 | EQ_SSS Assay3 | EQ_SSS Assay4 | Expected values |
| --- | --- | --- | --- | --- | --- | --- | --- |
| 1 | Ab2210_pos | 1 | 80 | 160 | 80 | 113 | 80-320 |
| 2 | Ab2210_pos | 2 | 160 | 160 | 80 | 113 | 80-320 |
| 3 | Ab2210_pos | 3 | 80 | 160 | 80 | 80 | 80-320 |
| 4 | Backtiter | 1 | 16 | 16 | 16 | 16 | 8-32 |
| 5 | Backtiter | 2 | 16 | 16 | 16 | 16 | 8-32 |
| 6 | Backtiter | 3 | 16 | 16 | 16 | 16 | 8-32 |
| 7 | IHRS 1:10 | 1 | 640 | 640 | 640 | 640 | 320-1280 |
| 8 | IHRS 1:10 | 2 | 640 | 640 | 640 | 640 | 320-1280 |
| 9 | IHRS 1:10 | 3 | 640 | 640 | 640 | 640 | 320-1280 |
| 10 | NR-31082_neg | 1 | 5 | 5 | 5 | 5 | <10 |
| 11 | NR-31082_neg | 2 | 5 | 5 | 5 | 5 | <10 |
| 12 | NR-31082_neg | 3 | 5 | 5 | 5 | 5 | <10 |

**Supplementary Table 2a: HAI assay extended qualification for H3N2 Influenza Strain A/Singapore/INFIMH-16-0019/2016, data on eleven curves generated for precision, accuracy, linearity and range testing.** GMTs by dilution, assay, and plate identifiers, for dilutions 1:10, 1:20, 1:40, 1:80. Column names identify the assay by notebook number, ending with the plate number from each respective assay.

| Obs | Dilution | EQ  PAL Assay1  P1 | EQ  PAL Assay1  P4 | EQ  PAL Assay2  P1 | EQ  PAL Assay2  P4 | EQ  PAL Assay3  P1 | EQ  PAL Assay3  P4 | EQ  PAL Assay4  P1 | EQ  PAL Assay4  P4 | EQ PAL Assay5  P1 | EQ  PAL Assay5  P4 | EQ PAL Assay6  P4 |
| --- | --- | --- | --- | --- | --- | --- | --- | --- | --- | --- | --- | --- |
| 1 | 1:10 | 640 | 640 | 640 | 640 | 640 | 640 | 1280 | 905 | 905 | 905 | 640 |
| 2 | 1:20 | 320 | 320 | 320 | 320 | 320 | 226 | 320 | 320 | 320 | 320 | 320 |
| 3 | 1:40 | 160 | 160 | 160 | 160 | 160 | 160 | 160 | 160 | 160 | 160 | 160 |
| 4 | 1:80 | 80 | 80 | 80 | 80 | 80 | 80 | 80 | 80 | 80 | 80 | 80 |

**Supplementary Table 2b: HAI assay extended qualification for H3N2 Influenza Strain A/Singapore/INFIMH-16-0019/2016, data on eleven curves generated for precision, accuracy, linearity and range testing.** GMTs by dilution, assay, and plate identifiers, for dilutions 1:160, 1:320, 1:640, 1:1280. Column names identify the assay by notebook number, ending with the plate number from each respective assay.

| Obs | Dilution | EQ  PAL Assay1  P2 | EQ PAL Assay1  P5 | EQ PAL Assay2  P2 | EQ  PAL Assay2  P5 | EQ  PAL Assay3  P2 | EQ  PAL Assay3  P5 | EQ  PAL Assay4  P2 | EQ PAL Assay4  P5 | EQ  PAL Assay5  P2 | EQ  PAL Assay5  P5 | EQ  PAL Assay6  P5 |
| --- | --- | --- | --- | --- | --- | --- | --- | --- | --- | --- | --- | --- |
| 1 | 1:160 | 40 | 40 | 40 | 40 | 40 | 40 | 40 | 40 | 40 | 40 | 40 |
| 2 | 1:320 | 20 | 20 | 20 | 20 | 20 | 20 | 20 | 20 | 20 | 20 | 20 |
| 3 | 1:640 | 10 | 10 | 10 | 10 | 10 | 10 | 10 | 10 | 10 | 10 | 10 |
| 4 | 1:1280 | 5 | 5 | 5 | 5 | 5 | 5 | 5 | 5 | 5 | 5 | 5 |

**Supplementary** **Table 2c: HAI assay extended qualification for H3N2 Influenza Strain A/Singapore/INFIMH-16-0019/2016, precision and accuracy results.** %GCV for intermediate precision and intra-operator repeatability, and %RE, by dilution.

| Obs | Dilution | %GCV IP | %GCV Intra-assay repeatability | %RE |
| --- | --- | --- | --- | --- |
| 1 | 1:10 | 25 | 11 | 17 |
| 2 | 1:20 | 10 | 10 | -3 |
| 3 | 1:40 | 0 | 0 | 0 |
| 4 | 1:80 | 0 | 0 | 0 |
| 5 | 1:160 | 0 | 0 | 0 |
| 6 | 1:320 | 0 | 0 | 0 |
| 7 | 1:640 | 0 | 0 | 0 |
| 8 | 1:1280 | 0 | 0 | 0 |

**Supplementary Table 3: HAI assay extended qualification for H3N2 Influenza Strain A/Singapore/INFIMH-16-0019/2016 linearity testing, percent linearity results.** The table depicts the data for pooled plasma in-house reference standard (IHRS), curves by assay and curve identifier, dilution, GMT at given dilution, previous dilution, and its GMT at previous dilution. The percent linearity is expected to be in the range of 50 and 200 percent. All observations meet this criterion.

| Obs | Assay Curve | Dilution | GMT | Previous Dilution | Previous dilution GMT | Percent Linearity |
| --- | --- | --- | --- | --- | --- | --- |
| 1 | EQ_PAL Assay1_C1 | 1:20 | 320 | 1:10 | 640 | 100 |
| 2 | EQ_PAL Assay1_C1 | 1:40 | 160 | 1:20 | 320 | 100 |
| 3 | EQ_PAL Assay1_C1 | 1:80 | 80 | 1:40 | 160 | 100 |
| 4 | EQ_PAL Assay1_C1 | 1:160 | 40 | 1:80 | 80 | 100 |
| 5 | EQ_PAL Assay1_C1 | 1:320 | 20 | 1:160 | 40 | 100 |
| 6 | EQ_PAL Assay1_C1 | 1:640 | 10 | 1:320 | 20 | 100 |
| 7 | EQ_PAL Assay1_C1 | 1:1280 | 5 | 1:640 | 10 | 100 |
| 8 | EQ_PAL Assay2_C1 | 1:20 | 320 | 1:10 | 640 | 100 |
| 9 | EQ_PAL Assay2_C1 | 1:40 | 160 | 1:20 | 320 | 100 |
| 10 | EQ_PAL Assay2_C1 | 1:80 | 80 | 1:40 | 160 | 100 |
| 11 | EQ_PAL Assay2_C1 | 1:160 | 40 | 1:80 | 80 | 100 |
| 12 | EQ_PAL Assay2_C1 | 1:320 | 20 | 1:160 | 40 | 100 |
| 13 | EQ_PAL Assay2_C1 | 1:640 | 10 | 1:320 | 20 | 100 |
| 14 | EQ_PAL Assay2_C1 | 1:1280 | 5 | 1:640 | 10 | 100 |
| 15 | EQ_PAL Assay3_C1 | 1:20 | 320 | 1:10 | 640 | 100 |
| 16 | EQ_PAL Assay3_C1 | 1:40 | 160 | 1:20 | 320 | 100 |
| 17 | EQ_PAL Assay3_C1 | 1:80 | 80 | 1:40 | 160 | 100 |
| 18 | EQ_PAL Assay3_C1 | 1:160 | 40 | 1:80 | 80 | 100 |
| 19 | EQ_PAL Assay3_C1 | 1:320 | 20 | 1:160 | 40 | 100 |
| 20 | EQ_PAL Assay3_C1 | 1:640 | 10 | 1:320 | 20 | 100 |
| 21 | EQ_PAL Assay3_C1 | 1:1280 | 5 | 1:640 | 10 | 100 |
| 22 | EQ_PAL Assay4_C1 | 1:20 | 320 | 1:10 | 1280 | 50 |
| 23 | EQ_PAL Assay4_C1 | 1:40 | 160 | 1:20 | 320 | 100 |
| 24 | EQ_PAL Assay4_C1 | 1:80 | 80 | 1:40 | 160 | 100 |
| 25 | EQ_PAL Assay4_C1 | 1:160 | 40 | 1:80 | 80 | 100 |
| 26 | EQ_PAL Assay4_C1 | 1:320 | 20 | 1:160 | 40 | 100 |
| 27 | EQ_PAL Assay4_C1 | 1:640 | 10 | 1:320 | 20 | 100 |
| 28 | EQ_PAL Assay4_C1 | 1:1280 | 5 | 1:640 | 10 | 100 |
| 29 | EQ_PAL Assay5_C1 | 1:20 | 320 | 1:10 | 905 | 71 |
| 30 | EQ_PAL Assay5_C1 | 1:40 | 160 | 1:20 | 320 | 100 |
| 31 | EQ_PAL Assay5_C1 | 1:80 | 80 | 1:40 | 160 | 100 |
| 32 | EQ_PAL Assay5_C1 | 1:160 | 40 | 1:80 | 80 | 100 |
| 33 | EQ_PAL Assay5_C1 | 1:320 | 20 | 1:160 | 40 | 100 |
| 34 | EQ_PAL Assay5_C1 | 1:640 | 10 | 1:320 | 20 | 100 |
| 35 | EQ_PAL Assay5_C1 | 1:1280 | 5 | 1:640 | 10 | 100 |
| 36 | EQ_PAL Assay6_C1 | 1:20 | 320 | 1:10 | 640 | 100 |
| 37 | EQ_PAL Assay6_C1 | 1:40 | 160 | 1:20 | 320 | 100 |
| 38 | EQ_PAL Assay6_C1 | 1:80 | 80 | 1:40 | 160 | 100 |
| 39 | EQ_PAL Assay6_C1 | 1:160 | 40 | 1:80 | 80 | 100 |
| 40 | EQ_PAL Assay6_C1 | 1:320 | 20 | 1:160 | 40 | 100 |
| 41 | EQ_PAL Assay6_C1 | 1:640 | 10 | 1:320 | 20 | 100 |
| 42 | EQ_PAL Assay6_C1 | 1:1280 | 5 | 1:640 | 10 | 100 |
| 43 | EQ_PAL Assay1_C2 | 1:20 | 320 | 1:10 | 640 | 100 |
| 44 | EQ_PAL Assay1_C2 | 1:40 | 160 | 1:20 | 320 | 100 |
| 45 | EQ_PAL Assay1_C2 | 1:80 | 80 | 1:40 | 160 | 100 |
| 46 | EQ_PAL Assay1_C2 | 1:160 | 40 | 1:80 | 80 | 100 |
| 47 | EQ_PAL Assay1_C2 | 1:320 | 20 | 1:160 | 40 | 100 |
| 48 | EQ_PAL Assay1_C2 | 1:640 | 10 | 1:320 | 20 | 100 |
| 49 | EQ_PAL Assay1_C2 | 1:1280 | 5 | 1:640 | 10 | 100 |
| 50 | EQ_PAL Assay2_C2 | 1:20 | 320 | 1:10 | 640 | 100 |
| 51 | EQ_PAL Assay2_C2 | 1:40 | 160 | 1:20 | 320 | 100 |
| 52 | EQ_PAL Assay2_C2 | 1:80 | 80 | 1:40 | 160 | 100 |
| 53 | EQ_PAL Assay2_C2 | 1:160 | 40 | 1:80 | 80 | 100 |
| 54 | EQ_PAL Assay2_C2 | 1:320 | 20 | 1:160 | 40 | 100 |
| 55 | EQ_PAL Assay2_C2 | 1:640 | 10 | 1:320 | 20 | 100 |
| 56 | EQ_PAL Assay2_C2 | 1:1280 | 5 | 1:640 | 10 | 100 |
| 57 | EQ_PAL Assay3_C2 | 1:20 | 226 | 1:10 | 640 | 71 |
| 58 | EQ_PAL Assay3_C2 | 1:40 | 160 | 1:20 | 226 | 141 |
| 59 | EQ_PAL Assay3_C2 | 1:80 | 80 | 1:40 | 160 | 100 |
| 60 | EQ_PAL Assay3_C2 | 1:160 | 40 | 1:80 | 80 | 100 |
| 61 | EQ_PAL Assay3_C2 | 1:320 | 20 | 1:160 | 40 | 100 |
| 62 | EQ_PAL Assay3_C2 | 1:640 | 10 | 1:320 | 20 | 100 |
| 63 | EQ_PAL Assay3_C2 | 1:1280 | 5 | 1:640 | 10 | 100 |
| 64 | EQ_PAL Assay4_C2 | 1:20 | 320 | 1:10 | 905 | 71 |
| 65 | EQ_PAL Assay4_C2 | 1:40 | 160 | 1:20 | 320 | 100 |
| 66 | EQ_PAL Assay4_C2 | 1:80 | 80 | 1:40 | 160 | 100 |
| 67 | EQ_PAL Assay4_C2 | 1:160 | 40 | 1:80 | 80 | 100 |
| 68 | EQ_PAL Assay4_C2 | 1:320 | 20 | 1:160 | 40 | 100 |
| 69 | EQ_PAL Assay4_C2 | 1:640 | 10 | 1:320 | 20 | 100 |
| 70 | EQ_PAL Assay4_C2 | 1:1280 | 5 | 1:640 | 10 | 100 |
| 71 | EQ_PAL Assay5_C2 | 1:20 | 320 | 1:10 | 905 | 71 |
| 72 | EQ_PAL Assay5_C2 | 1:40 | 160 | 1:20 | 320 | 100 |
| 73 | EQ_PAL Assay5_C2 | 1:80 | 80 | 1:40 | 160 | 100 |
| 74 | EQ_PAL Assay5_C2 | 1:160 | 40 | 1:80 | 80 | 100 |
| 75 | EQ_PAL Assay5_C2 | 1:320 | 20 | 1:160 | 40 | 100 |
| 76 | EQ_PAL Assay5_C2 | 1:640 | 10 | 1:320 | 20 | 100 |
| 77 | EQ_PAL Assay5_C2 | 1:1280 | 5 | 1:640 | 10 | 100 |

**Supplementary Table 4: HAI assay extended qualification for H3N2 Influenza Strain A/Singapore/INFIMH-16-0019/2016, specificity testing and response calls.** GMT and response call (negative or positive), for controls and samples tested for specificity, are shown in this table, by sample description, and assay identifier. All samples met the expected response calls.

| Obs | Sample Description | Response | EQ Spe Assay1 | EQ Spe Assay2 |
| --- | --- | --- | --- | --- |
| 1 | FR-1000 | Geomean_titer | 5 | 5 |
| 2 | FR-1000 | response_call | Negative | Negative |
| 3 | FR-1250 | Geomean_titer | 5 | 5 |
| 4 | FR-1250 | response_call | Negative | Negative |
| 5 | FR-1487 | Geomean_titer | 2560 | 2560 |
| 6 | FR-1487 | response_call | Positive | Positive |
| 7 | FR-1562 | Geomean_titer | 2560 | 2560 |
| 8 | FR-1562 | response_call | Positive | Positive |
| 9 | FR-1612 | Geomean_titer | 2560 | 2560 |
| 10 | FR-1612 | response_call | Positive | Positive |
| 11 | FR-1613 | Geomean_titer | 5 | 5 |
| 12 | FR-1613 | response_call | Negative | Negative |
| 13 | FR-1682 | Geomean_titer | 5 | 5 |
| 14 | FR-1682 | response_call | Negative | Negative |
| 15 | FR-1683 | Geomean_titer | 2560 | 2560 |
| 16 | FR-1683 | response_call | Positive | Positive |
| 17 | FR-1685 | Geomean_titer | 5 | 5 |
| 18 | FR-1685 | response_call | Negative | Negative |
| 19 | Normal Goat | Geomean_titer | 5 | 5 |
| 20 | Normal Goat | response_call | Negative | Negative |

**Supplementary Table 5: Study design for qualification and extended qualification, where In-house reference standard was used for testing precision, accuracy, linearity, range, LOD, LOQ.** The tables show the counts of assays, scientists and number of GMTs produced within each assay, for the respective dilution series used for that testing and analysis. For extended qualification a more balanced study design was used, such that the full range of 8 dilutions was tested across all six assays, to increase the number of observations for analysis.

| Qualification | | | | | | | | |
| --- | --- | --- | --- | --- | --- | --- | --- | --- |
| **Precision, accuracy**  **(1:20, 1:80, 1:320, 1:640)** | | | | | Total | **Linearity, range, LOD, LOQ**  **(1:10, 1:20, 1:40, 1:80, 1:160, 1:320, 1:640, 1:1280)** | | Total |
| **Assay** | 1 | 2 | 3 | 4 | 4 | 1 | 2 | 2 |
| **Scientist** | A | B | A | B | 2 | A | B | 2 |
| **GMT per assay** | 5 | 2 | 2 | 2 | 11 × 4 dilutions = 44 | 1 | 1 | 2 × 8 dilutions = 16 |

| Extended qualification | | | | | | | |
| --- | --- | --- | --- | --- | --- | --- | --- |
| **Linearity, precision, accuracy, range, LOD, LOQ**  **(1:10, 1:20, 1:40, 1:80, 1:160, 1:320, 1:640, 1:1280)** | | | | | | | Total |
| **Assay** | 1 | 2 | 3 | 4 | 5 | 6 | 6 |
| **Scientist** | A | B | A | B | A | B | 2 |
| **GMT per assay** | 2 | 2 | 2 | 2 | 2 | 2* | 11 × 8 dilutions = 88 |
| *1 set of data was excluded due to experimental error. | | | | | | | |
